# Negative SARS-CoV-2 PCR or rapid antigen test result and the subsequent risk of being infectious: a mathematical simulation study

**DOI:** 10.1186/s12874-021-01361-3

**Published:** 2021-08-10

**Authors:** Ralf Krumkamp, Benno Kreuels, Veronika K. Jaeger, Jürgen May, Rafael Mikolajczyk, André Karch

**Affiliations:** 1 Department of Infectious Disease Epidemiology, Bernhard Nocht Institute for Tropical Medicine, Bernhard Nocht Str. 74, 20359 Hamburg, Germany; 2 German Center for Infection Research (DZIF), Partner site Hamburg - Lübeck - Borstel – Riems, Hamburg, Germany; 3 Department of Tropical Medicine, Bernhard Nocht Institute for Tropical Medicine, Bernhard Nocht Str. 74, 20359 Hamburg, Germany; 4 I. Department of Medicine, University Medical Center Hamburg-Eppendorf, Martinistraße 52, 20251 Hamburg, Germany; 5 Department of Medicine, College of Medicine, P. O. Box 278, Zomba Blantyre, Malawi; 6 Institute of Epidemiology and Social Medicine, University of Muenster, Albert-Schweitzer-Campus 1, 48149 Münster, Germany; 7 Tropical Medicine II, University Medical Centre Hamburg-Eppendorf, 20151 Hamburg, Germany; 8 Institute for Medical Epidemiology, Biometrics and Informatics, Interdisciplinary Center for Health Sciences, Martin Luther University Halle-Wittenberg, Magdeburger Straße 8, 06112 Halle, Germany

**Keywords:** SARS-CoV-2, Negative diagnostic tests, Infectiousness, Asymptomatic transmission

## Abstract

**Background:**

A considerable proportion of SARS-CoV-2 transmission occurs from asymptomatic and pre-symptomatic cases. Therefore, different polymerase chain reaction (PCR)- or rapid antigen test (RAT)-based approaches are being discussed and applied to identify infectious individuals that would have otherwise gone undetected. In this article, we provide a framework to estimate the time-dependent risk of being infectious after a negative SARS-CoV-2 test, and we simulate the number of expected infectious individuals over time in populations who initially tested negative.

**Methods:**

A Monte Carlo approach is used to simulate asymptomatic infections over a 10-days period in populations of 1000 individuals following a negative SARS-CoV-2 test. Parameters representing the application of PCR tests or RATs are utilized, and SARS-CoV-2 cumulative 7-day incidences between 25 and 200 per 100,000 people are considered. Simulation results are compared to case numbers predicted via a mathematical equation.

**Results:**

The simulations showed a continuous increase in infectious individuals over time in populations of individuals who initially tested SARS-CoV-2 negative. The interplay between false negative rates of PCR tests or RATs, and the time that has passed since testing determines the number of infectious individuals. The simulated and the mathematically predicted number of infectious individuals were comparable. However, Monte Carlo simulations highlight that, due to random variation, theoretically observed infectious individuals can considerably exceed predicted case numbers even shortly after a test was conducted.

**Conclusions:**

This study demonstrates that the number of infectious individuals in a screened group of asymptomatic people can be effectively reduced, and this effect can be described mathematically. However, the false negative rate of a test, the time since the negative test and the underlying SARS-CoV-2 incidence are critical parameters in determining the observed subsequent number of cases in tested population groups.

**Supplementary Information:**

The online version contains supplementary material available at 10.1186/s12874-021-01361-3.

## Background

A considerable proportion of individuals with SARS-CoV-2 infection are free of symptoms or only show very mild symptoms. However, transmission can occur from asymptomatic, pre-symptomatic and from symptomatic infections [1, 2]. Different polymerase chain reaction (PCR)- or rapid antigen test (RAT)-based approaches are currently being considered or have already been implemented to identify asymptomatic infections that would have otherwise gone undetected (for example, to protect clinically vulnerable individuals in high-infection risk settings like nursing homes, to reduce unnecessary quarantine of non-infectious people, or to lift social contacts restrictions e.g., to permit care home visiting, travelling, restaurant visits or leisure time activities) [3].

For such measures to be effective, laboratory tests would ideally be done in real-time, as the test result reflects the current state of infectiousness of an individual. Since this is not always possible, especially for PCR diagnostics, tests done within a certain time frame are accepted. In travel restrictions, this time frame is usually 48 h before travel [4]. In contrast, RAT results are available within 15–30 min. However, RATs have a lower sensitivity than PCR tests [5, 6]. The time between sample taking and the event of interest is a crucial parameter in determining the individual risk of being infectious. An individual who has tested negative may be in the latent period of infection at sampling and could progress to an infectious state immediately thereafter. As time since testing increases, individuals with a previous negative test result will have the same risk of being infectious as the underlying population.

In this article, we provide a framework to estimate the time-dependent risk of being infectious after a negative PCR test or RAT, and we simulate the number of expected infectious individuals over time in example groups of individuals who had a negative test result.

## Methods

**Fig. 1:**
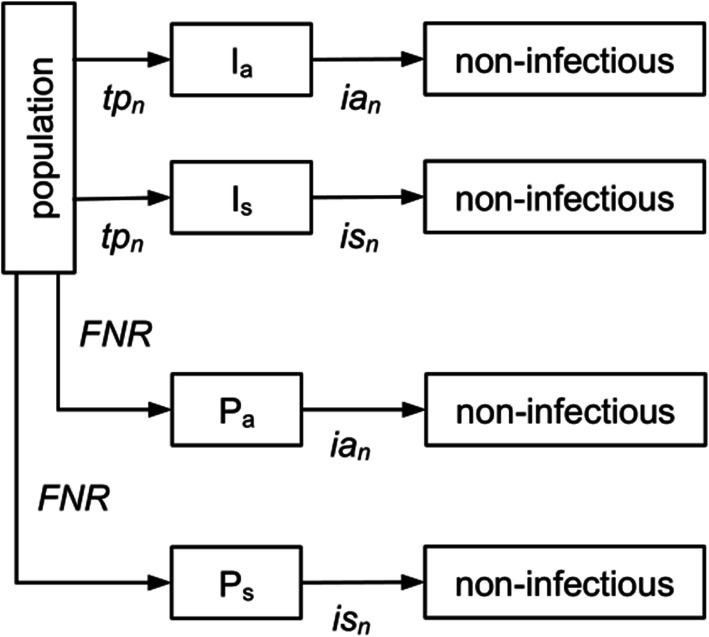
State chart of the simulation model, representing the states of infections an individual can go through. Abbreviations: *FNR*, false positive rate; *tp*_*n*_, individual start of the infectious period; *ia*_*n*_, individual duration of the infectious period for asymptomatic infections; *is*_*n*_, individual duration of the infectious period for symptomatic infections

We simulate the number of infectious individuals over time present in a group of people who tested negative for SARS-CoV-2 at *t*_*0*_. The model is designed to represent a group of people that was tested as a prerequisite to join an event, in order to estimate the number of infections over time which would have gone undetected. The model is individual-based, and a simulated person can change from non-infectious to infectious to non-infectious within time periods randomly selected from disease-specific distributions. Figure 1 shows the flow of individuals through the respective disease states. Two groups of infectious individuals are simulated in the models, namely (i) prevalent infections in individuals who falsely tested negative at the simulation start (*t*_*0*_), and (ii) incident infections, occurring after the simulation start. Furthermore, the model differentiates between asymptomatic infections (individuals who stay in the population and may potentially infect others) and symptomatic infections (individuals who show symptoms after a pre-symptomatic infectious period, after which they isolate themselves and are no longer able to infect others). The duration of infectiousness of an individual with an asymptomatic course of infection (*ia*_*n*_, infectious period of individual *n*) is sampled from a gamma distribution, with alpha = 4 and beta = 1.25. The pre-symptomatic infectious period of an individual with asymptomatic course of infection (*is*_*n*_, pre-symptomatic infectious period of individual *n*) is sampled from a gamma distribution, with alpha = 4 and beta = 0.525. These parameter settings are based on values used by Davis et al. [7, 8] in SARS-CoV-2 mathematical modelling studies. The model is set up as follows:

(i) Missed prevalent infections: A population of 1000 individuals is established, within which infections occur over a 20-day period and from which asymptomatic prevalent infections are selected. Infectious individuals are randomly determined (binomial distribution) according to the cumulative 7-day incidence. About 35% of all SARS-CoV-2 cases are expected to be symptomatic and the remaining 65% show only very mild or no symptoms [9]; these proportions are used to allocate each infectious individual to one of these groups (binomial distribution). The time when infectiousness starts (*tp*_*n*_, start of infectious period of individual *n*) is allocated to each infectious individual, following a uniform distribution. The duration of the infectious period is added to this time-point, sampled from the distribution of the pre-infectious period (for individuals who become symptomatic) or the complete infectious period (for individuals who remain asymptomatic). All individuals who are within their infectious period at the end of this initial simulation step form the group of prevalent asymptomatic infectious cases. The false negative rate (*FNR*) of a test defines the proportion of infectious individuals with a negative test result, i.e., the proportion of prevalent infectious individuals missed at the simulation start. Thus, from the simulated prevalent infections a proportion, as defined by *FNR*, is randomly selected (binomial distribution) as prevalent infections at *t*_*0*_. The respective remaining infectious period for each individual defines the time when that individual becomes non-infectious.

(ii) Incident infections: In the simulation step above, a group of 1,000 individuals was established, of which some are prevalent infectious individuals at *t*_*0*_. Individuals non-infectious at *t*_*0*_ can become infectious during the next step of the simulation. Infectious individuals that occur over time are determined according to the cumulative 7-day incidence following a binomial distribution. The time at which infectiousness starts (*tp*_*n*_) and the duration of infectiousness (*is*_*n*_ or *ia*_*n*_, for symptomatic or asymptomatic course of infection, respectively) is allocated to each infected individual, as described above. The simulation is applied for a period of 10 days.

The final simulation model contains prevalent infectious individuals at *t*_*0*_ and incident infections occurring after *t*_*0*_, each individual becoming non-infectious when its infectious period ends. Finally, the number of infectious individuals present over time is summarised. As our calculations focus on the current number of infectious cases, any kind of incubation or latent period is not considered in the model. For the simulations we assume that non-infectious individuals are entirely susceptible with no immunity, are not suspected of being positive for SARS-CoV-2, and new cases are expected to occur randomly (i.e., unclustered).

Models in which all individuals received either a PCR test or a RAT are calculated. Parameters are taken from the literature as summarised in Table 1. PCR tests and RATs differ in their *FNR*s. *FNR*s depend on the sensitivity of a test and the accuracy of the test implementation. Reported *FNR*s for SARS-CoV-2 PCR tests vary greatly [10] and 3% was used as base case value. For RATs, the World Health Organization recommends minimum performance requirements of 80% sensitivity [6], which was assumed for calculations of the *FNR* in the RAT scenarios. Viral loads rise quickly at the beginning of the infectious period. However, in the first 12 h after a PCR test is able to detect an infection, high circle threshold (ct) values are observed (i.e., low viral loads) and cases are assumed to be non-infectious during this time interval [11, 12]. Hence, we determined that during the first 12 h (Gaussian distribution; mean = 12, standard deviation (SD) = 1) after an infected individual would be detectable by PCR, the individual would be non-infectious. For both tests, simulations with reported cumulative 7-day incidences of 25, 50, 100 and 200 per 100,000 people were calculated. The cumulative 7-day incidences, as reported by surveillance systems, primarily capture symptomatic SARS-CoV-2 cases. However, as outlined above, only 35% of all SARS-CoV-2 infections are expected to be symptomatic [9]. To estimate the actual number of infectious individuals for the simulated populations, the assumed incidences (as reported by surveillance systems) are corrected and divided by the proportion of symptomatic cases. Since absolute numbers of infections in the model population will be small, the Monte Carlo method was applied to show stochastic effects on the occurrence of infectious individuals. Simulations are repeated 2,000 times using parameter distributions as outlined above. In order to summarise Monte Carlo results, the number of infectious cases over time in the simulation runs was tabulated using a one-hour intervals.

**Table 1:** Parameters applied in the Monte Carlo simulations

Parameter	Value	Reference
Reported SARS-CoV-2 incidences	25, 50, 100 & 200 /100,000 per 7 days	
Proportion symptomatic infections	0.35	[9]
*FNR* for PCR test	3%	[10]
*FNR* for RAT	20%	[6]
Complete infectious period	Gamma (*alpha* = 4, *beta* = 1.25)	[7, 8]
Pre-symptomatic infectious period	Gamma (*alpha* = 4, *beta* = 0.525)	[7, 8]
Mean infectious periods	*alpha* * *beta*	
Time between PCR positivity and infectiousness	Mean = 12 h (SD: 1)	[11, 12]
Model group size	1000	
Model runs	2000	

*Abbreviations*: *FNR* False negative rate, *PCR* Polymerase chain reaction, *RAT* Rapid antigen test, *SD* Standard deviation;

The expected proportion of asymptomatic infectious cases over time (*t*) within a population which had a negative SARS-CoV-2 test at *t*_*0*_ can be described mathematically. Prevalent infections becoming non-infectious and newly occurring incident infections eventually becoming non-infectious are calculated. Calculations are based on the corrected daily incidence of infectious individuals (*Ic*, cumulative 7-day incidence divided by 7 over the proportion of symptomatic cases). A proportion (defined in *FNR*) of prevalent asymptomatic infectious cases remains undetected after a diagnostic test. Prevalent asymptomatic infections (*P*) are calculated by *Ic* times the mean infectious period, which equals *alpha* * *beta*, the parameters from the gamma distribution defining the length of the infectious period. Since different distributions for symptomatic and asymptomatic infections are applied, prevalence for each group must be calculated separately. Prevalent infections become negative when the infectious period ends, and this reduction over time is estimated with the cumulative distribution functions (CDF) of the respective infectious periods. The proportion 1-*Pr*(X ≤ *t* * 2) remains infectious at *t,* while *t* is multiplied by 2 because the infectious period began before *t*_*0*_ and, on average, half of this time is already depleted. Equation 1 and 2 describe the reduction of prevalent infectious individuals over time for symptomatic infections (respective parameters denoted with subscript *s*) and asymptomatic infections (respective parameters denoted with subscript *a*). In a population in which all individuals had a negative SARS-CoV-2 test at *t*_*0*_, new infections emerge successively. The number of infections increases continuously over time until the first people reach the end of their infectious periods and become non-infectious. Thus, as soon as the last infected individuals complete their infectious periods, the number of newly emerging infectious individuals and the number of infectious individuals becoming non-infectious is balanced (i.e., steady state). In eq. 3 and 4, Ic * t describes the increase of new infectious individuals over time for symptomatic (*s*) and asymptomatic infections (1-*s*). Simultaneously, infected individuals become non-infectious when their infectious period ends. This is estimated by the accumulated CDFs for symptomatic and asymptomatic infections, respectively, which define the number of incident cases that become non-infectious between *t*_*0*_ und *t*.

1 $$ {P}_s\ast FNR\ast \left(1-{\mathit{\Pr}}_s\left(X\le t\ast 2\right)\right) $$

2 $$ {P}_a\ast FNR\ast \left(1-{\mathit{\Pr}}_a\left(X\le t\ast 2\right)\right) $$

3 $$ Ic\ast s\ast t-\sum \limits_{i=1}^t Ic\ast t\ast {\mathit{\Pr}}_s\left(X\le i\right) $$

4 $$ Ic\ast \left(1-s\right)\ast t-\sum \limits_{i=1}^t Ic\ast t\ast {\mathit{\Pr}}_a\left(X\le i\right) $$

Results of these 4 equations are summed to estimate the prevalence of asymptomatic and pre-symptomatic infections at *t*. Where PCR test scenarios are calculated, the 12-h offset to infectiousness must be considered; thus, if 0 < *t* ≤ 12 h, *t* = 0, else *t* = *t*-12*.* In this calculation, homogeneous infection occurrence is assumed.

The numbers of infectious individuals from the Monte Carlo simulations are compared to mathematically predicted numbers calculated by the equation derived above. The mean numbers over time for the respective Monte Carlo scenarios are calculated. The equation is then applied to calculate the expected numbers over time using simulation parameters and both results are displayed using line graphs. All calculations were done in R version 4.0.3 [13].

## Results

**Fig. 2:**
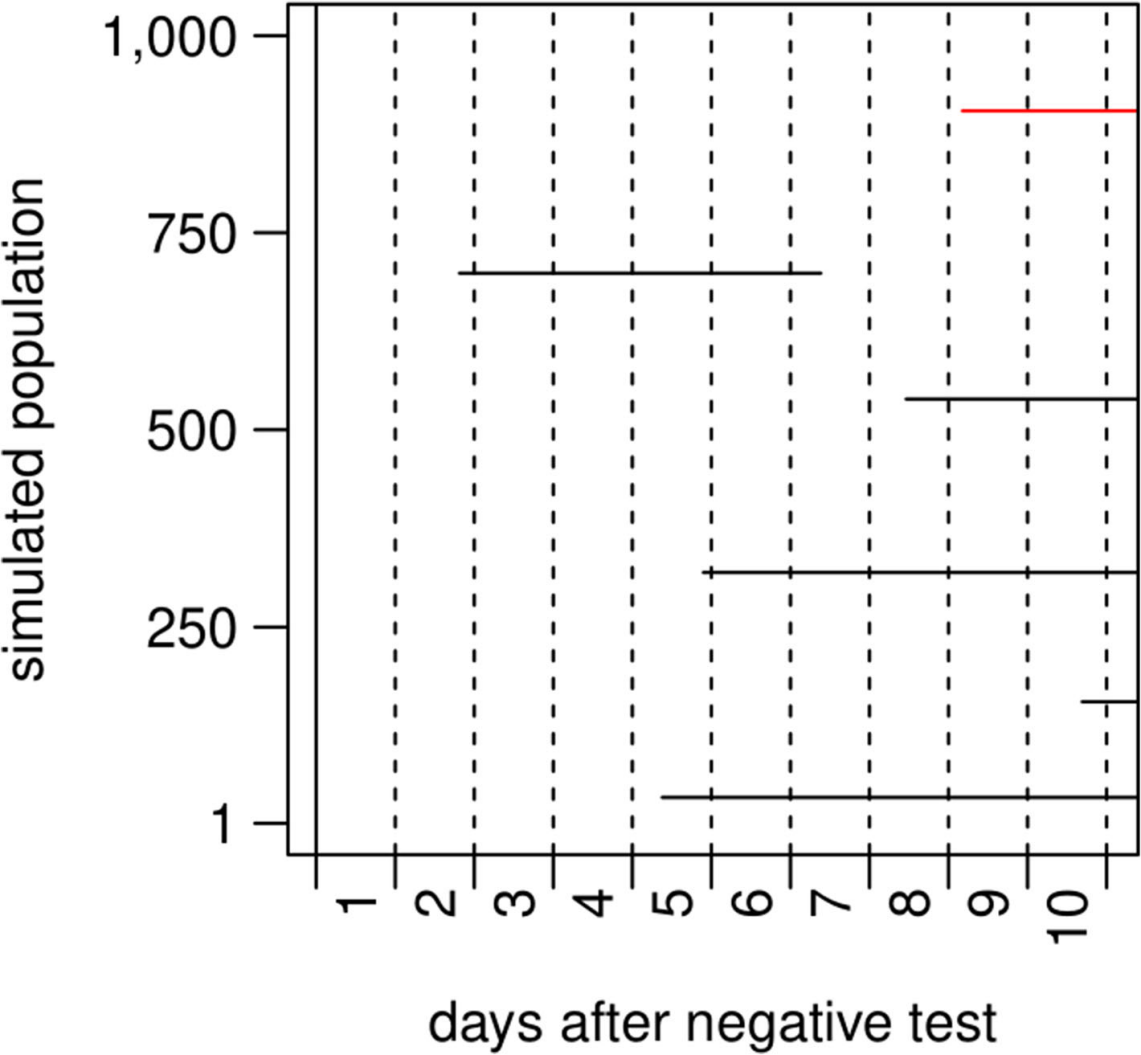
Example of a single baseline model, showing simulated infectious periods in a population of 1000 individuals over 1 week

To visualise the occurrence of infectious individuals within a previously SARS-CoV-2 negative population, a simple baseline simulation was established (Fig. 2). The simulation represents a population of 1,000 individuals who were tested with RATs, assuming a reported cumulative 7-day incidence of 100 cases per 100,000 people. The horizontal lines in Fig. 2 show infectious periods that emerged over ten days. In total, 6 infections occurred during the simulation. The first infection occurred 44 h after the simulation start and the highest number of infectious individuals was observed at the end of day 10, when 5 individuals were infectious simultaneously. One of the infectious individuals is pre-symptomatic (red line) and this individual would be removed from the simulated population with the start of disease symptoms. However, the end of the infectious period is outside the simulated period.

**Fig. 3:**
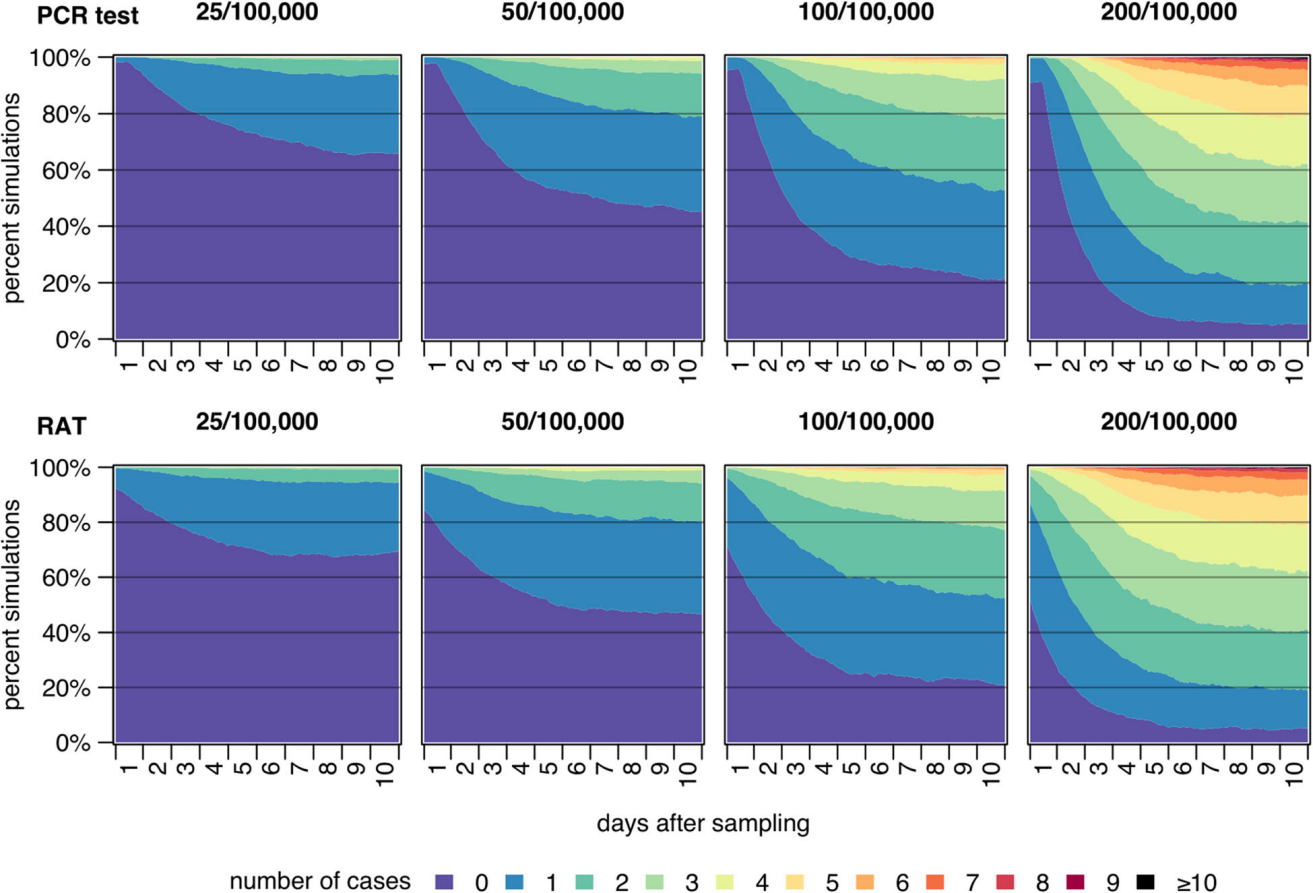
Percentages of simulations with different number of symptomatic and pre-symptomatic infections calculated using Monte Carlo method, considering different scenarios in a population of 1000 individuals. Abbreviations: PCR, polymerase chain reaction; RAT, rapid antigen test

To capture stochastic effects, the Monte Carlo method was employed and simulations were repeated 2,000 times based on the scenarios outlined above. Figure 3 summarises the results of the Monte Carlo simulations using area graphs, where the proportion of calculated simulations is shown on the y-axis. The number of infectious individuals over time after a negative SARS-CoV-2 test observed in the simulations is indicated by the coloured areas over the x-axis. Graphs in the first row show simulation results of the PCR test, and those in the second row of the RAT strategy. The graphs summarise simulation results based on different reported cumulative 7-day incidences over time. When assessing the PCR-based strategy, infectious cases occurred as early as at *t*_*0*_ due to the assumed *FNR* of 3% for PCR tests. No infectious individuals were observed at the start in 98% of simulations when the reported cumulative 7-day incidence was 25/100,000, in 98% of simulations when incidence was 50/100,000, in 95% when incidence was 100/100,000, and in 91% when incidence was 200/100,000. After one day (24 h), no infectious individuals had occurred at the indicated incidence levels in 94, 89, 79 and 63% of simulations, and after 2 days (48 h) in 86, 73, 53 and 30% of the simulations. Eventually, by the end of the simulation period (10 days), no infectious individuals occurred in 66, 45, 21 and 6% of the simulations, which represents distributions in non-selected populations. As time progressed, multiple infections were likely to occur in scenarios with higher cumulative incidences. More than 4 infectious individuals were observed after 115 h, 84 h, 49 h, and 21 h in simulations based on cumulative 7-day incidences of 25, 50, 100 and 200 cases per 100,000 people, respectively.

The *FNR* of the RAT was set to 20%, resulting in a lower proportion of simulations without any infectious individuals at *t*_*0*_ in the RAT-based strategy. No infectious cases were observed at the start of the simulations in 92, 85, 71 and 52% of the simulations at incidence levels of 25, 50, 100 and 200 per 100,000, respectively. No infectious individuals had occurred after 1 day (24 h) in 86, 72, 54 and 28% of simulations, and after 2 days (48 h) in 80, 63, 41 and 16% of the simulations, respectively. No infectious individuals had occurred by the end of the simulation period in 69, 46, 21 and 5% of the simulations, comparable to the numbers calculated by the PCR scenarios. In simulations based on a reported cumulative 7-day incidence of 25/100,000, no simulation showed more than 4 cases; however, at cumulative 7-day incidences of 50, 100 and 200/100,000, more than 4 cases were observed after 53 h, 1 h, and 3 h, respectively.

**Fig. 4:**
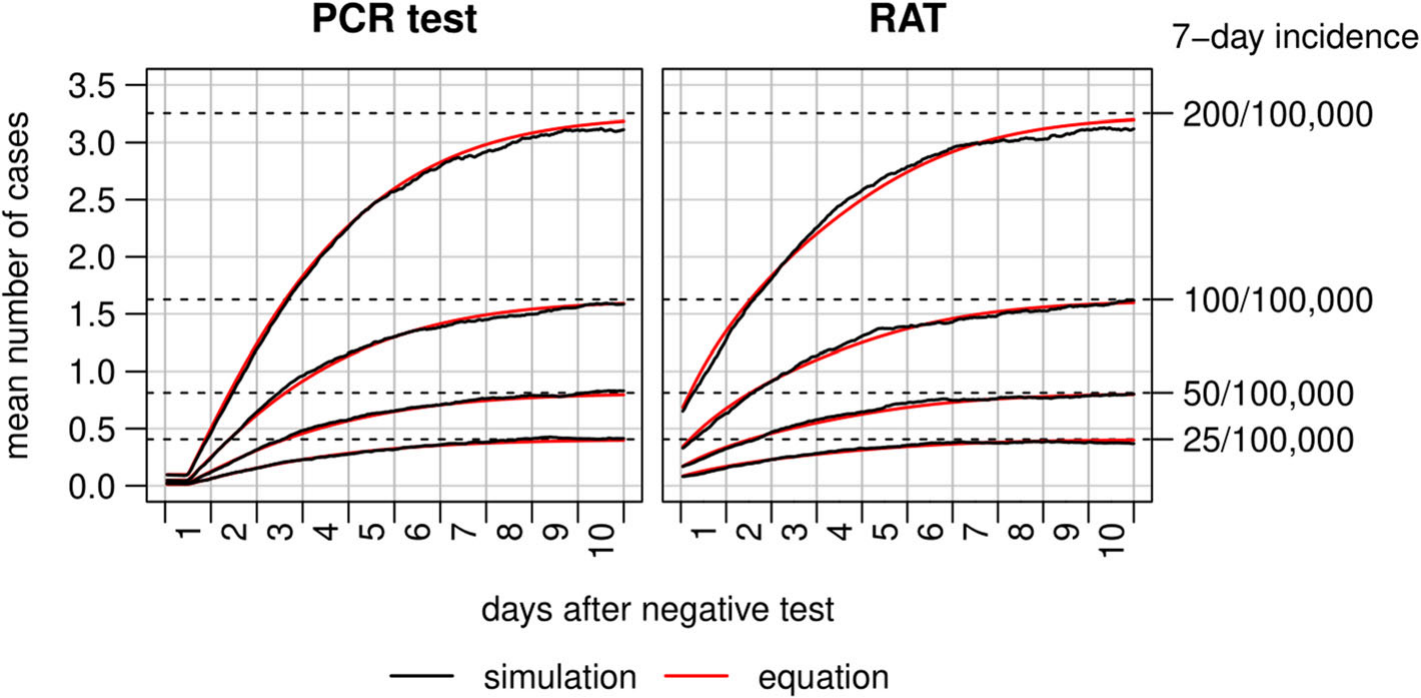
Number of asymptomatic and pre-symptomatic individuals over time in a group of 1000 people averaged over the Monte Carlo simulation scenarios (black lines) and estimated by the equation as derived in the method section (red lines). Abbreviations: PCR, polymerase chain reaction; RAT, rapid antigen test

To compare simulation-based and mathematically derived case numbers, the mean number of infectious individuals over time per scenario was calculated. The equation derived in the method section was applied to calculate the expected case numbers over time using the respective simulation parameters. Figure 4 shows calculated numbers from the PCR test (first plot) and the RAT scenarios (second plot). The mean numbers of infectious individuals from the simulations are shown by the black lines, and the estimated numbers calculated with the equation by the red lines. The numbers of infectious individuals calculated by both methods show a high agreement. In both graphs, a curved increase in infections is observed until the end of the simulation period and the steady state situation, indicated by the dashed lines, is nearly reached. An infected individual is detectable by PCR test as early as 12 h before onset of infectiousness, which is why numbers remain constant at the beginning of the simulation periods in the first graph.

## Discussion

Our analysis shows that asymptomatic testing with PCR tests or RATs can be applied to establish population groups with a temporarily minimized number of infectious individuals, for example for events requiring a safe bubble for participants. We provide a mathematical formula to estimate the expected number of infectious individuals over time using the disease incidence, the time of the infectious period and the time that has passed since a test was administered. However, Monte Carlo simulations performed in our analysis highlight how, due to random variation, observed infectious individuals can considerably exceed these expected numbers even shortly after all individuals in the population had tested negative. In a fraction of simulated scenarios, single infectious individuals occurred right after the simulation start; in high-incidence simulations, even multiple cases were present at the same time. These results are important for infection control because they demonstrate that, while testing can be used to effectively select groups of people with a low number of infectious, infectious individuals may emerge immediately after the population had tested negative, especially at high incidences.

PCR tests are superior to RATs in terms of their *FNR* [5] and their ability to identify infections even before individuals are infectious [11, 12]. In contrast, RATs can be applied in non-laboratory settings and results are available within 30 min. For PCR tests, the delay between test administration and reporting of results is often 24 to 48 h. Hence, the risk of a false negative test result and the delay between sample collection and result reporting must be considered when interpreting any test result. If the high FNR of RATs is to be compensated for by a shorter delay in result reporting, it is important that the test is carried out immediately before a critical event takes place. The higher FNR still bears the risk of missing infections, but the immediate application better estimates the individual’s real-time infection status, reducing the number of infectious individuals which may emerge before the event of interest takes place.

Simplifying assumptions that were made to illustrate principles of SARS-CoV-2 testing should be considered when interpreting the results. Random infection occurrence in tested populations was assumed; however, SARS-CoV-2 is reported to spread in clusters and via super-spreading events [14]. Thus, in the case that testing is performed on a group of people where a superspreading event occurred (e.g., residents and personnel of a nursing facility with an ongoing SARS-CoV-2 outbreak), the prevalence of infectious individuals would be considerably higher compared to the used numbers inferred from the cumulative 7-day incidences. The proportion of infectious individuals without or with only mild symptoms was set to 65%. However, a correct estimate of this proportion is subject to several methodological limitations that make interpretation of the reported frequencies of symptoms among SARS-CoV-2 cases difficult [15]. Additionally, the likelihood of showing symptoms of an infection with SARS-CoV-2 is associated with factors like age [16] or with different underlying health conditions [17], which again can differ among tested groups. We assume that the remaining 35% of cases are recognized by a health care system and that they contribute to the observed incidence at population level. These figures are context-dependent and subject to current testing strategies. However, for many health care systems, a lower proportion should be assumed. In the literature, there are conflicting reports about infectious periods in asymptomatic, mild, moderate or severe symptomatic infections as well as among different age-groups [18]. Furthermore, we did not consider the application of any containment strategies, like quarantine of traced contacts or of household members of identified individuals. However, measures may further reduce the number of infectious persons attending an event.

SARS-CoV-2 control strategies based on testing of asymptomatic individuals are suggested and designed for different purposes. A model of SARS-CoV-2 outbreaks in long-term care facilities evaluated the ability of different testing strategies to identify ongoing transmission early. The authors highlight that expanding surveillance beyond symptom-based screening could allow for earlier outbreak detection; however, testing strategies must consider available testing capacities [19]. Another study modelled the effect of surveillance testing to control SARS-CoV-2 transmission, concluding that asymptomatic individuals should be considered in testing strategies. Effective surveillance depends largely on the frequency of testing and the speed of reporting, and is only marginally improved by high test sensitivity [9]. Routine testing strategies for SARS-CoV-2 infection to facilitate safe airline travel and to mitigate the spread of the virus was analyzed in a simulation study. The authors concluded that this can be an effective strategy to reduce passenger risk of infection during travel, although measures to reduce population-level transmission should be in place when travelling from a high to low incidence setting [20]. In contrast, we focus on the question of being asymptomatic and infectious after a negative diagnostic test. In other words, how long does a negative test result provide some safety for people attending an event. Our analysis shows the dependence between the time at which a negative test was performed and the time individuals are infectious, with the distribution of the infectious period being a key parameter in this context.

## Conclusions

Our analysis highlights the temporal dynamics of SARS-CoV-2 infections after a negative test within a theoretical population. We show that PCR tests or RATs can be used to select a group of people with a reduced number of SARS-CoV-2 infections. However, the parameters representing time since testing negative and the underlying SARS-CoV-2 incidence in a population are critical in determining the expected number of infectious individuals in groups of people who initially tested negative. Thus, especially in high-incidence scenarios, additional infection control measures are still needed to reduce transmission risk from undetected infectious individuals.

## Supplementary Information

**Additional file 1.** R-code of the Monte Carlo simulation model.

## Data Availability

The R-code of the Monte Carlo simulation model is available as Additional file 1.

## Abbreviations

CDF: Cumulative distribution function
Ct: Circle threshold
*FNR*: False negative rate
*ia*_*n*_: Infectious period of individual *n*
*Ic*: Corrected daily incidence of asymptomatic and pre-symptomatic infections
*is*_*n*_: Pre-symptomatic infectious period of individual *n*
*P*: Prevalence of asymptomatic and pre-symptomatic infections
PCR: Polymerase chain reaction
RAT: Rapid antigen test
*s*: Symptomatic infection
SARS-CoV-2: Severe acute respiratory syndrome coronavirus 2
SD: Standard deviation
*t*: Time point
*tp*_*n*_: Start of infectious period of individual *n*
